# Turtle genomic novelty is driven by the evolution of ecological robustness

**DOI:** 10.1016/j.isci.2026.114975

**Published:** 2026-02-11

**Authors:** Jule Drewalowski, Yuejiao Huang, Wessel Mulder, David A. Duchêne

**Affiliations:** 1 Department of Biology, Faculty of Science, University of Copenhagen, 2100 Copenhagen, Denmark; 2 Globe Institute, Faculty of Health and Medical Sciences, University of Copenhagen, 1350 Copenhagen, Denmark; 3 Center for Macroecology Evolution and Climate, Globe Institute, University of Copenhagen, Copenhagen, Denmark; 4 The Environment Institute, School of Biological Sciences, University of Adelaide, Adelaide, SA 5005, Australia; 5 Department of Public Health, University of Copenhagen, 1353 Copenhagen, Denmark

**Keywords:** Zoology, Molecular biology, Evolutionary biology

## Abstract

Turtles are striking among vertebrates in their unique diversity of morphology, ecology, and life-history. A robust physiology is often seen as the primary driver of turtle evolution, and this is increasingly studied in comparative genomics frameworks. Here, we use a genomic-scale dataset from across turtles and a novel comparative method to examine the genomic forces driving evolution in the group. Our approach addresses the correlation structures across species and genes flexibly via Mahalanobis distances from gene-wise pairwise genetic matrices, relaxing the assumption of an overarching species phylogeny. Analysis of outliers in this space reveals that close relatives, notably Cryptodira and Emydidae, share the most accelerated genes, and functional enrichment further indicates that these genes are involved in stress tolerance and immunity. These results reinforce ecological robustness as a core adaptation in turtles, and point to neutral processes as contributing to the genes with accelerated evolution in animals.

## Highlights

• Shared turtle traits are frequently unlinked with jumps in numbers of fast evolving genes
• Numbers of shared accelerated genes are conserved, suggesting low rates in this form of novelty
• The appearance of accelerated genes in turtles follows a roughly constant rate through time
• Turtles share accelerated rates in genes for stress tolerance, immunity and gene regulation

## Graphical abstract

**Figure:**
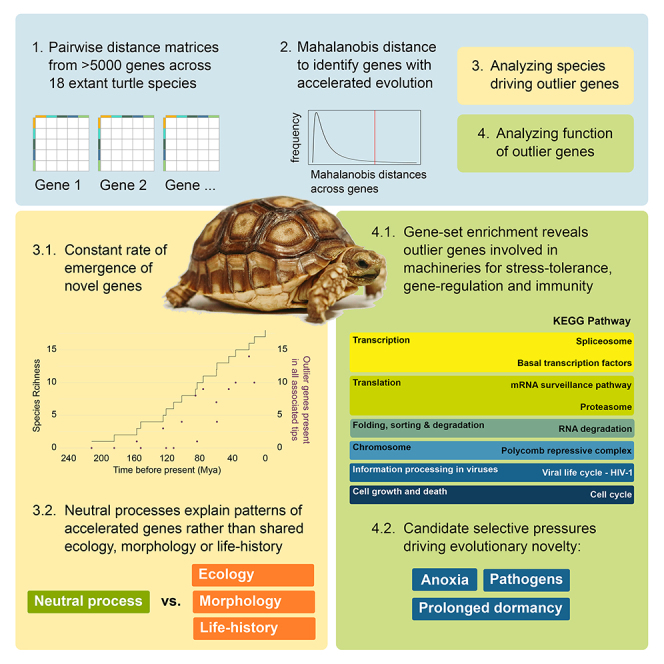

## Introduction

Turtles (order Testudines) are a monophyletic group of reptiles with ancient origins in the Permian period^1^ (>260 Mya). They are widely known for their robust carapace and longevity, but are now undergoing fast decline due to human activity.^2^ Turtle diversity is also represented in adaptations to a myriad of habitats,^3^ diverse life-history strategies,^4^ home range sizes,^5^ and migratory cycles.^6^ Testudines are divided into two major groups based on their neck retraction mechanism; the Pleurodira (side-necked turtles), and the Cryptodira (hidden-necked turtles) which include most living tortoises and turtles, including the large Galápagos tortoise, sea turtles and the large, diverse Emydidae family.^7^

Genomic-scale data are increasingly used to make robust inferences on the placement of turtles in the Tree of Life,^8^ and have revealed some of the genomic pathways that explain their primary adaptations across habitats. For instance, accelerated evolutionary rates for oxidative phosphorylation genes in sea turtles are proposed as a mitochondrial molecular adaptation to stress, resulting from the more active lifestyle of these marine dwellers.^9^ In the threatened olive ridley turtle, adaptive changes in genes linked to olfaction, vision, virus defense, and longevity are proposed as the genetic underpinning of adaptation to the marine environment.^10^ However, the roles of accelerated genomic regions and relative importance of ecology in driving genomic novelty across turtles remains largely unstudied, and biased toward few charismatic marine-dwelling groups. Data for a set of species representative of the entire turtle lineage can provide a window into the ecological and genetic drivers of novelty across all turtles, by testing evolutionary hypotheses in a comparative genomics framework.^11^

The field of comparative genomics is increasingly revealing the drivers of genomic innovations across the Tree of Life, shedding light on major forces of evolution in turtles and other vertebrate groups. For instance, non-collinearity in immune- and sensory-related gene families between green turtles and leatherbacks has been proposed to underlie their contrasting ecological strategies, occupancy of shallow coastal versus open-ocean habitats, differences in diet, and patterns of reproductive philopatry.^12^ Work on large numbers of avian genomes has revealed a signal of substantial evolution in song, ossification,^13^ energy usage,^14^ and gene regulation,^15^ and similar insights are now possible across diverse vertebrate groups.^13^^,^^16^ Extending genomic analysis to large numbers of taxa and genomes involves practical and methodological challenges arising from the inference of evolutionary rates, the assumption of a known phylogeny, and the difficulty in disentangling processes across large numbers of genes and taxa.

One of the primary challenges in comparative genomics is to identify the relative contributions of lineages and genes, respectively, to variation in evolutionary rates.^17^^,^^18^ Anomalous genes in subsets of lineages can be extracted using several methodological frameworks, usually assuming a known or co-inferred phylogeny.^19^^,^^20^ These interactions between genes and lineages can reveal outlier genetic elements with unique metabolic or genomic functions that have had a major influence on evolution.^21^ However, accurately identifying the lineages and genes with substantial evolutionary change is complicated by the need to balance phylogenetic non-independence among lineages, phylogenetic inference error, and the differences among gene genealogies due to incomplete lineage sorting.^20^^,^^22^^,^^23^ Hence, there is a need for novel and efficient phylogeny-free comparative methods that flexibly adjust for correlation structures in genomic-scale datasets.

Using a genomic-scale dataset from 18 species of turtles^16^ representing all major Testudine clades, we examine the metabolic pathways and lineages with accelerated evolutionary rate. To identify these genes, we propose a highly flexible and computationally efficient approach for exploring differences in molecular evolution across lineages in genomic-scale datasets. Specifically, we model genetic pairwise distances calculated from each gene as a multivariate distribution (see STAR Methods). Mahalanobis distances can then be calculated across genes or taxa to identify those with unusually fast evolution, while accounting for the correlation structures in the data which might arise from factors like age since divergence. These correlation structures are not based on pre-defined phylogenetic data, making the method phylogeny-free, and instead express any joint variance caused by evolutionary rates and gene-tree discordance. The analyses were repeated using a phylogeny-aware method, expected to have reduced power to reveal molecular evolutionary change.^23^ We then use gene set enrichment analysis to test the hypothesis that outlier genes play joint metabolic roles, related with ecological adaptation or morphology. By focusing on genes across functional regions, we aim to identify the evolutionary processes shaping genomes of Testudines, further elucidating the evolutionary trajectory of this unique group.

## Results and discussion

### Relatedness drives shared accelerated genes in turtles

Data from 5,084 protein-coding genes in 18 extant species of turtles were used for inferring synonymous (*d*_S_) and non-synonymous (*d*_N_) pairwise distance matrices (see STAR Methods). Genes and lineages with large outlier *d*_S_ are expected to have accelerated mutation rates, while those with outlier *d*_N_ are likely additionally impacted by selection and population size,^24^ assuming weak codon-usage bias.^25^ The ratio between the two values (*d*_N_/*d*_S_; *ω*) is thus driven primarily by selection and population size. If outlier accelerated genes are driven by morphology or ecology, then taxa with similar biology might have overlap in the genes with *ω* falling above the 5% upper tail of the complete distribution. These genes can be considered as accelerated relative to the mean genomic speed. For instance, aquatic habitats or shell fusion might be expected to be linked with substantial overlap across taxa in genes with outlier *ω*.

**Figure 1:**
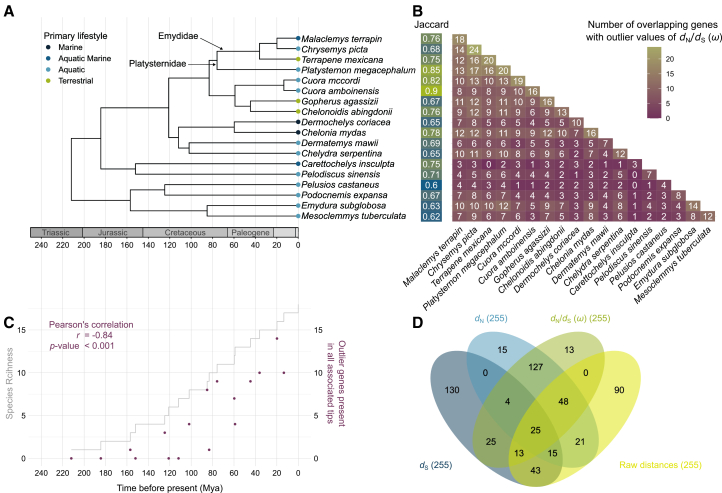
Quantity and taxonomic distribution of genes with outlier *ω* showing overlap across turtle groupings (A) Dated phylogenetic tree of the turtle species included in this study adapted from Thomson et al. (2021)^28^ and primary lifestyle from Wang et al. (2025).^27^ (B) Jaccard similarities in accelerated genes across taxa and heatmap showing the number of detected outlier genes overlapping between species pairs. (C) Relationship between phylogenetic divergence times (*x* axis) and lineages through time (left *y* axis), with the sum of the number of genes across descendant tips for each node (right *y* axis). The Pearson’s correlation describes the correlation between the outlier genes present in descendant taxa (right *y* axis) and divergence time (*x* axis). (D) Overlapping genes determined as outliers in each of the multivariate distributions for *d*_N_, *d*_S_, *ω*, and overall molecular distances.

Our analyses show that genes with outlier high *ω* in turtles are primarily shared among closer relatives (Figure 1B). This is in contrast with the hypothesis that overlaps in outlier genes are driven by ecology or morphology, such that accelerated genes might converge across distant relatives. Instead, we find younger taxa like Emydid terrapins have large overlap in outlier genes, also substantially overlapping with their sister *Platysternon megacephalum*, a species within the monotypic family Platysternidae that has been traditionally difficult to place in the phylogeny of turtles.^26^ While *P. megacephalum* is ecologically and morphologically distinct from many Emydids, it shares a similar pace of life to the Emidid genus *Terrapene*, with smaller, less frequent clutches.^27^ The broad diversity in life-history, ecology and morphology among these relatives sharing genes with outlier *ω* suggest that gene acceleration is primarily associated the novelty that is shared among related lineages.

Older lineages of turtles have lower numbers of genes with accelerated evolution compared with more closely related counterparts (Figure 1). Nonetheless, exceptions include *Chelonia mydas*, *Chelydra serpentina*, and *Emydura subglobosa*, which have large numbers of outlier genes that show considerable overlap with Emydidae and Platysternidae, as well as with one another, despite being widely diverse in their ecology, morphology and life-history strategies. *C. mydas*, occupies marine environments while *C. serpentina* and *E. subglobosa* occupy freshwater habitats, including rivers, ponds, and lakes. *C. serpentina* and *C. mydas* share migratory behavior, longer lifespans, late maturity and large, infrequent clutches, whereas *E. subglobosa* has a shorter lifespan, with smaller, more frequent clutches.^27^ Notably, *C. mydas* shares many more outlier genes with smaller, freshwater turtles than with taxa with more pronounced similarities in their habitats, migratory nature and body size, like the leatherback sea turtle *Dermochelys coriacea*. Additionally, *E. subglobosa* shares more outlier genes with Emydidae and Playsternidae, despite being placed in the distantly related subfamily of Pleurodira, the side-necked turtles. The other representatives of this distant group from Emydids, *Pelusios castaneus*, *Podocnemis expansa*, and *Mesoclemmys tuberculata* show more overlap with the young taxon of Cryptodira, hidden-necked turtles, than with one another.

A multi-response phylogenetic regression revealed no significant relationship between genomic uniqueness of each species and their ecological, morphological or life-history traits (Figure S1). This indicates that species-level distinctiveness in accelerated gene evolution is not explained by any of these traits. Taken together, our results point toward a predominantly phylogenetic signature in the distribution of genes under apparent selection, with diminishing overlap among increasingly ancestral taxon pairs.

The genes with outlier synonymous genetic distances (*d*_S_) are mostly a non-overlapping set from the genes with outlier non-synonymous distances (*d*_N_; Figure 1D). The differences in results between *d*_S_ and *d*_N_ is expected if the two metrics are driven by distinct evolutionary forces. Specifically, this result is likely to reflect the impact of silent changes in *d*_S_ with respect to amino acid change, reflecting mostly neutral evolution. Also consistent with this result is the finding in further downstream analyses that *d*_S_ outlier genes are not associated with any over-represented metabolic pathways, as expected if the metric is primarily selectively neutral.

### Molecular clock in the emergence of outlier genes

Instead of a clear association with morphology or ecology, we find the overlap among taxa in genes with outlier *ω* to have an inverse link with divergence times. Specifically, our data appear to show an approximately monotonous drop in overlapping genes with increasing divergence times (Pearson’s *r* = −0.838, *p* value <0.001; Figure 1C). One explanation is that neutral processes, such as population size fluctuations, drive the accumulation of changes that lead to some genes becoming important ecologically, as defined by an accelerated *ω*. This would imply that a mechanism resembling a molecular clock dictates the accumulation of novel genes with accelerated selective evolution, or positive selection. This could also indicate that neutral theory of molecular evolution extends to impact genome-wide processes of metabolic pathway evolution,^29^ as opposed to only impacting evolution at the nucleotide level.

Alternatively, emergence of new outlier genes could be linked with the process of diversification, with limited detectable major shifts in these dataset due to the small sample size and relatively constant accumulation of lineages (Figure 1C). Previous work has suggested that nucleotide evolution is linked with diversification rates in a wide range of taxa,^30^ and that punctuated change can be seen upon fast events of diversification.^31^ Similarly to nucleotide evolution, it could be that the acceleration of groups of ecologically relevant genes is linked with punctuated acceleration in diversification rates. This is consistent with estimates of fast diversification in some turtle groupings, such as those including Emydidae and Platysternidae.^16^ Research on birds has shown major functional evolutionary changes at times of fast diversification,^15^ and studies on other taxonomic groups will help to reveal whether the phenomenon is widespread.

One pervasive issue in comparative analysis of molecular evolution is the node-density effect, whereby great sampling in taxon-rich groups leads to the detection of additional molecular changes.^32^^,^^33^ Our method partially bypasses this effect by allowing for independent inferences of pairwise distances among taxa, which are not constrained by a phylogenetic tree structure. Analysis of pairwise distance matrices can furthermore be consistent under the multi-species coalescent, and can allow for non-treelike processes such as recombination and introgression, even within genes.^34^ However, deep hybridization events might still have similar signatures in distance matrices to major shifts in evolutionary rates,^23^ and it is difficult to identify events in time and space without a phylogenetic estimate. Still, pairwise distance matrices offer a flexible window into shared evolution among taxa, and this can be explored further via testing the function of outlier genes.

### Stress-tolerance gene evolution in turtles

**Figure 2:**
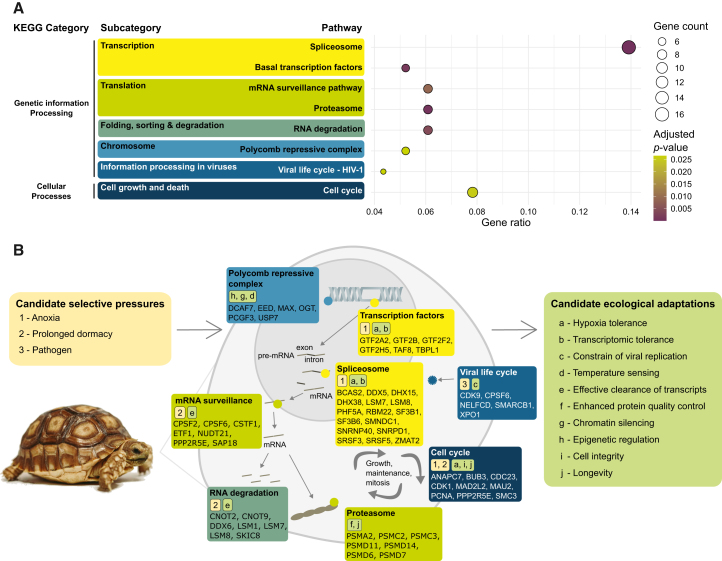
Metabolic pathway enrichment results for genes identified as fast-evolving outlier *ω* evolution (A) The eight significantly KEGG enriched pathways varied widely in their gene ratio support, and (B) covered a broad range of cellular processes associated with ecological robustness. For each enriched pathway candidate selective pressures and candidate ecological adaptations are listed, as well as contributing outlier genes.

To characterize accelerated genes with outlier *ω,* we performed KEGG enrichment analysis on the genes identified as outliers, falling beyond the upper 5% of the multivariate distribution of distances. We found eight significantly enriched pathways among these genes, segregating into two main functional classes: genetic information processing, and cellular processes (Figure 2A; Table S1). One of the enriched terms was the core cell-cycle machinery (Figure S2A), which is the primary pathway for orchestrating cell division, including DNA replication and chromosome segregation. This pathway plays a crucial role in balancing cellular repair and maintenance, particularly during physiological stressful events of dormancy, hibernation or oxygen deprivation.^35^ Our results likely reflect accelerated evolution in mechanisms modulating the cell cycle, including arrest at low-activity periods (e.g., *G*_0_/*G*_1_), to reduce DNA damage, conserve energy, and lower metabolic demands.^35^ Regulated cell-cycle arrest is a common adaptive strategy among ectotherms experiencing prolonged environmental stress.^36^^,^^37^ Accelerated evolution in the cell-cycle machinery in turtles might reflect selection for enhanced cellular integrity and longevity. Analyses using phylogeny-aware comparative methods provided no evidence of enriched genetic pathways in outlier genes, as expected if there is a loss of power to identify molecular change when assuming an overarching phylogenetic correlation structure.^23^

The enrichment in HIV-1 viral life cycle pathway (Figure S2B) reflects selection pressures on the cellular machinery that are frequently hijacked by viruses broadly, rather than being specific to Lentiviruses like HIV-1. This result of accelerated evolution in immunity-related genes is consistent with work on western painted turtles that has revealed rapid expansion of innate immune gene families, including Toll-like receptors.^38^ Similarly, the elevated *ω* that we observe in genes such as XOP1, CDK9, CPSF6, NELFCD, and SMARCB1 likely reflects lineage-specific adaptations in nuclear trafficking, transcriptional elongation, RNA processing and chromatin remodeling to constrain viral replication. The evolution of pathogen resistance has been proposed as a foundational form of adaptation, and our data supports exposure to pathogens as a fast-evolving machinery that likely had a central role in turtle evolution and diversification.

Additional enriched pathways in turtle genes with outlier accelerated evolutionary rates included the spliceosome, basal transcription factors, mRNA surveillance, the proteasome, RNA degradation, and the polycomb repressive complex (PRC) (Figures 2A and S2). Collectively, these pathways constitute ribonuclear, proteostatic, and epigenetic machineries governing mRNA processing, protein turnover, and chromatin regulation. In turtles, the evolution of spliceosomal and basal transcription components has been associated with transcriptomic plasticity and hypoxia tolerance during anoxia, while selection on mRNA surveillance and RNA degradation machinery facilitates efficient clearance of aberrant transcripts during prolonged dormancy.^39^^,^^40^ Similarly, positive selection on proteasome-ubiquitin genes aligns with enhanced proteostasis and longevity in long-lived vertebrates, suggesting improved protein quality control that has likely contributed to the extended lifespans observed in turtles.^41^ The enrichment of outlier genes in PRC pathways further indicates lineage-specific adaptations in epigenetic regulation, potentially influencing developmental arrest and temperature-dependent sex determination. This hypothesis is also supported in previous work on *Lepidochelys olivacea*, where JARID2 was found to recruit proteins from the PRC2 complex (e.g., EFD) to imprint H3K27me3 marks at sex determination pathways, linking chromatin silencing to environmental temperature sensing.^42^ Together, these findings point to a role of accelerated evolution of fundamental gene regulation machineries in shaping the unique physiological and developmental adaptations of turtles.

Our findings are consistent with previous reports of selective pressures on genes related to longevity mechanisms and immune regulation in marine turtles^10^^,^^12^ and western painted turtles.^38^ Unlike these prior studies, our analysis did not recover enrichment in sensory pathways related to vision, olfaction, and natal homing. Selective pressures for anoxia-response genes have previously been reported in multiple species of Emydidae, such as the western painted turtle and the red-eared slider,^43^ which are known to tolerate long and pronounced periods of extreme cold and anoxia during hibernation. In addition to these previously uncovered pathways, we present evidence of selective pressure acting on pathways related to temperature sensing. This may represent a general condition for ectotherms, for which behavioral thermoregulation is critical for physiology and survival, even during the embryonic stage.^44^ Further work on larger datasets will build further evidence of whether this genomic signature is turtle-specific or shared across other ectotherms.

Our findings revealing accelerated change in the spliceosome, basal transcription factors, mRNA surveillance highlight the importance of gene regulation machinery in driving evolutionary change, which is consistent with patterns observed in other vertebrate radiations such as birds.^15^ This signal from these pathways with large numbers of genes appears despite our focus being restricted to a subset of conserved genes (BUSCOs), emphasizing the potential strength and relevance of these genes in comparative analyses. A possible caveat is that KEGG pathway enrichment compares a selected gene set, in this case BUSCO outliers, against a broader background of all annotated genes. This can introduce bias if other background genes that are absent from our analysis play key roles in relevant biological processes. Nonetheless, we repeated analyses using a soft-shelled turtle genome as the background set, which led to identical results to analyses with the green sea turtle as reference (Figure S3A). A reference encompassing the full set of data across four turtle species as the background consistently revealed the previously mentioned eight enriched pathways, but with an additional three pathways related to neurodegenerative disease, e.g., Huntington disease, spinocerebellar ataxia and amyotrophic lateral sclerosis (Figure S3B). We propose that these pathways are possible false positives that arise from the reduced number of background genes and power associated with pooling multiple reference genomes.

The overall low numbers of outlier genes observed along less diverse lineages, with more ancient divergence times among the sampled taxa, may reflect lower genomic rates of molecular evolution in those taxa, or may be attributed to the set of genes analyzed. The use of BUSCO genes, while beneficial for ensuring broad taxonomic coverage and high-quality alignments, inherently biases the dataset toward highly conserved, single-copy orthologs. These genes may be less likely to capture lineage-specific adaptive changes, particularly those linked to unique ecological, morphological, or life-history traits. Furthermore, our filtering criterion—retaining only gene trees with data from at least nine species—may exclude genes with rare occurrence across clades, possibly filtering out signatures of adaptations that are not widely shared across turtles. In future studies, the relationships between ecological, morphological, or life-history characteristics and rates of molecular evolution may be more apparent when adopting a broader set of genes or less stringent filtering criteria.

### Mahalanobis distances prioritize function-enriched outlier genes

To verify the validity of the new phylogeny-free method, we compared analyses with those using a phylogeny-aware method, implemented in ClockstaRX.^20^ Specifically, we tested the hypothesis that the two methods identify similar sets of outlier genes. We find that the genes found to be fast evolving by the two methods have greater overlap than expected by chance (Figure S4; Fisher’s exact test of overlap *p* values <0.001). The significant overlap in results between two methods indicates that they are extracting similar signals from the data. However, the absolute portion of overlapping genes between two sets is consistently around 10% of each set, allowing for substantial deviation in the enriched gene functions identified by these two methods.

Strikingly, gene set enrichment analyses on the outlier genes from phylogeny-aware methods do not support any enriched KEGG pathways, suggesting that our Mahalanobis approach has greater power in this framework. Traditional phylogeny-based comparative methods incorporate a pre-defined phylogenetic structure as driving non-independence among data points, which risks a shift of molecular change from ancient gene-specific branches toward the present.^23^ Meanwhile, our phylogeny-free approach absorbs any form of correlation structure that is present in the data, flexibly addressing any variation in phylogenetic non-independence across lineages and genes. Nonetheless, our method relies on distance matrices, which inherently involve a loss of information about molecular change at the sequence level.^45^ Our use of distance matrices also incorporates large amounts of non-independent measures and possibly redundancy in the signals across the data, complicating statistical interpretation of the results. Further extensions to this method will continue to improve the power in evolutionary comparative genomics.

Using an efficient approach for molecular evolutionary analysis based on pairwise molecular distances, we show that genes with accelerated evolution are primarily shared by close relatives. This points to a role for neutral processes—possibly enabled by population level or diversification processes—as drivers of gene acceleration across deep timescales. Our data also show that ecological robustness has been an important evolutionary force in genomic evolution across turtles, with an important role in the evolution of metabolic conservation, immune responses, and epigenetic control and plasticity. Robustness and resilience has long been assumed to be a defining characteristic of the turtle lineage, and this work raises some of the genomic underpinnings of that hypothesis. Future work on more comprehensive sets of genes is likely to shed further light on the primary genomic adaptations of turtles, and combined with efficient methods of genomic analysis will reveal the major driving forces for evolution across the Tree of Life.

### Limitations of the study

The approach proposed can suffer from non-independence between data points and does not explicitly incorporate timescale in inferences of evolutionary rates, despite relaxing assumptions about bifurcating evolutionary history. Similarly, the dependence of BUSCO gene sets is widespread and likely to bias downwards the number of genes identified and studied in analyses of species with limited well-annotated relatives. Similarly, gene set enrichment analyses focus on well-established genetic pathways that might themselves evolve, such that our analyses might not incorporate many rare metabolic and molecular pathways. Future improvements to our work will involve extensions to these databases and analysis frameworks, and this progress will likely be aided by techniques in artificial intelligence.

## Resource availability

### Lead contact

Further information and requests may be directed to and will be fulfilled by the lead contact Wessel Mulder (bhr597@sund.ku.dk). David A. Duchene (david.duchene@sund.ku.dk) can also be contacted for further information.

### Materials availability

This study did not generate new unique reagents.

### Data and code availability

• This paper analyzes existing, publicly available data, accessible at https://doi.org/10.5061/dryad.tdz08kq14.
• All original code has been deposited at github.com/wessel-mulder/genomic-novelty and is publicly available at as of the date of publication.
• Any additional information required to reanalyze the data reported in this paper is available from the lead contact upon request.

## Acknowledgments

D.A.D. is funded by a 10.13039/501100009708Novo Nordisk Fonden Emerging Data Science Investigator Award (NNF23OC0084647). We thank all three anonymous reviewers for their comments and suggestions.

## Author contributions

J.A.D., Y.H., and W.M. conceived the study, and performed data collection, data preparation, and analyses of molecular data. All authors contributed to the design of analyses, data interpretation, and drafting the manuscript.

## Declaration of interests

The authors declare no competing interests.

## STAR★Methods

### Key resources table

**Table:** 

REAGENT or RESOURCE	SOURCE	IDENTIFIER
**Deposited data**		

Gene sequences across 18 species of turtles	Gable et al.^16^	https://doi.org/10.5061/dryad.tdz08kq14

**Software and algorithms**		

MACSE v2.07	Ranwez et al.^46^	https://www.agap-ge2pop.org/macse/
PAML v4.8	Yang^47^	http://abacus.gene.ucl.ac.uk/software/paml.html
seqkit v2.9.0	Shen et al.^48^	https://bioinf.shenwei.me/seqkit/
R v4.5.1	R Core Team^49^	https://www.R-project.org/
clusterProfiler v4.6.2	Yu^50^	https://bioconductor.org/packages/release/bioc/html/clusterProfiler.html
pathview v1.38.0	Luo and Brouwer^51^	https://bioconductor.org/packages/release/bioc/html/pathview.html
ape v5.8-1	Paradis et al.^52^	https://cran.r-project.org/web/packages/ape/index.html
tidyverse v2.0.0	Wickham et al.^53^	https://cran.r-project.org/web/packages/tidyverse/index.html
ggvenn v0.1.10	Wickham^54^	https://cran.r-project.org/web/packages/ggvenn/index.html
reshape2 v1.4.4	Wickham^55^	https://cran.r-project.org/web/packages/reshape2/index.html
ggtree v3.16.0	Yu et al.^56^	https://bioconductor.org/packages/release/bioc/html/ggtree.html
deeptime v2.2.0	Gearty^57^	https://cran.r-project.org/web/packages/deeptime/index.html
phangorn v2.12.1	Schliep^58^	https://cran.r-project.org/web/packages/phangorn/index.html
fishualize v0.2.3	Schiettekatte et al.^59^	https://cran.r-project.org/web/packages/fishualize/index.html
svglite v2.2.1	Wickham et al.^60^	https://cran.r-project.org/web/packages/svglite/index.html
MASS v7.3-61	Venables and Ripley^61^	https://cran.r-project.org/web/packages/MASS/index.html
KofamKOALA	Aramaki et al.^62^	https://www.genome.jp/tools/kofamkoala/
ClockstaRX v1.1.1	Duchêne et al.^20^	https://github.com/duchene/ClockstaRX
phytools v2.4-4	Revell^63^	https://cran.r-project.org/web/packages/phytools/index.html
Brms 2.23.0	Bürkner^64^	https://cran.r-project.org/web/packages/brms/index.html
Code for analysis		https://github.com/wessel-mulder/genomic-novelty

### Method details

Data were obtained from a recent phylogenomic study^16^ for 5,245 multiple sequence alignments (MSAs) of tetrapod BUSCO genes^65^ for 18 turtle species (Figure S5). We only kept genes that were available for at least nine of the represented turtle species, leading to 5,086 genes. As the original study did not perform codon-aware alignment or trimming, we decided to perform additional filtering and trimming steps to improve reliability of molecular distances among taxa. To ensure alignments were in the correct reading frame, we removed between zero and two additional bases from the beginning of the alignments, evaluating that alignments for all genes ending in a stop codon. Any internal stop codons were filtered out using MACSE.^46^ Furthermore, we excluded complete codons if a site contained gaps for >50% taxa, and where amino acid heterozygosity was >50%. We then filtered out genes with low information content, defined as those that contained less than ten parsimony informative sites. This final set of filtered MSAs included 5,085 BUSCO genes. Pairwise genetic distances among taxa for each gene were first calculated as simple raw Hamming distances. In addition, pairwise synonymous (*d*_S_) and non-synonymous (*d*_N_) pairwise distances were estimated using the universal genetic code and a F3x4 model of codon frequencies, as implemented in PAML.^47^ After removing one gene with insufficient information for inference of all distance matrices, the final set consisted of 5,084 genes with cophenetic and *ω* pairwise distance matrices for each.

#### Mahalanobis outlier gene detection

We propose that identifying outlier deviations in evolutionary rates in subsets of lineages and subsets of genes can be viewed as a test of molecular pairwise distances in multivariate space. Specifically, our starting point is a set of multiple sequence alignments including *N* taxa, for each of *i* genes. From the processed MSAs, we calculate the pairwise distance matrices among taxa within a gene, leading to *i* matrices of size *N* x *N*. The focus on pairwise distance matrices allows for examining the full data set of size *i* x *N* x *N*, for outliers in multivariate space. We then stack these pairwise distances to take Mahalanobis-distances across one dimension (e.g., genes). These distances account for possible correlation within and across matrices assuming multivariate error, and allow us to identify outliers of the dimension examined (i.e., genes or taxa). The focus on pairwise distances does not allow for examining ancient events as done in a full phylogenetic framework, and can obscure some of the nuances of the molecular substitution process. However, this data type allows for reduced assumptions about evolutionary history,^66^ which can complicate analyses in the face of discordance among gene trees.^23^ Our approach is flexible to widespread discordance among gene trees, non-treelike processes, and various metrics of molecular evolution such as cophenetic, amino acid, and of synonymous and non-synonymous change.

To identify outlier genes, we first collapsed the *i* x *N* x *N* three-dimensional matrix into a two-dimensional *i* x (*N* x *N)* matrix. In this format, each row represents a gene, and each column corresponds to a pairwise comparison among taxa, equivalent to a collapsed upper triangle of the distance matrix. From this matrix, Mahalanobis distances were calculated per gene, such that the correlation structure among taxa, for instance in relatedness, is considered to identify the 95th quantile of genes with the largest deviations from the overall distribution (Figures S6 and S7). For the genes identified as outliers, *p*-values were calculated using a *Χ*^2^ test based on the Mahalanobis distances, with the number of taxon pairwise comparisons as degrees of freedom and a Bonferroni adjustment for multiple comparisons.

Next, the set of outlier genes was re-analyzed to identify the specific taxa driving the outlier behavior of these genes. This analysis used a similar Mahalanobis-distance-based approach. The original *i* x *N* x *N* three-dimensional matrix was subset to include only the outlier genes *i*_outlier_ and then collapsed into a two-dimensional (*i*_outlier_ x *N*) x *N* matrix by stacking the full pairwise-distance matrices. In this case, the Mahalanobis distance is that among a given taxon at a given gene (rows), considering the possible correlation structure among taxa (columns). The 5% with greatest Mahalanobis distance were taken to be outliers, with their *p*-values calculated using a *Χ*^2^ null distribution with the number of taxa as degrees of freedom and a Bonferroni adjustment for multiple comparisons (Figure S6).

Our approach requires molecular pairwise distance matrices of equal size across genes. For gene matrices that did not include every pairwise combination of species, missing values were imputed using the mean of all pairwise distances for that specific gene and species-species combination. Mahalanobis assumes a multivariate normal distribution, such that the mean collapses unknown data to the centre of the distribution. It is unlikely that this leads to strong bias at the distribution extremes, the part of the distribution of interest. Imputation was always before matrix transposition to maintain data set size, and before log transformation to maintain data set scale. To identify the species driving these outlier genes, the entire matrix was used and thus, values in the diagonal are also imputed. Each diagonal value was calculated by taking the mean of the values for that species in that gene. Analyses were run using pairwise distance matrices derived from gene-trees, and rerun for the *d*_N_, *d*_S_ and *d*_N_/*d*_S_ ratio (*ω*) values, with log-transformed genetic distance matrices before Mahalanobis outlier detection analyses.

As a measure of genomic uniques, we calculate an average Jaccard index for each species by taking the mean of all pairwise Jaccard indices involving that species. We ran a multi-response phylogenetic regression to identify any correlations between genomic uniqueness and traits related to species habitats, life-history and morphology derived from the CheloniansTraits database^27^ (Figure S1). We also looked at the amount of shared outlier genes between all pairs of two taxa based on *d*_N_, *d*_S_, *d*_N_/*d*_S_ and raw Hamming genetic distance (Figure 1C; Figures S8–S11).

#### Comparison to a phylogeny-aware method

In order to validate our new method against a traditional phylogeny-aware approach, we used the branch-wise PCA method implemented in ClockstaRX^20^ as an alternative for identifying outlier genes. We adapted the published dated turtle species tree from Thomson et al. (2021)^28^ and used the pairwise *ω* distances to build least-squares branch length estimates per locus, under the species tree topology. Permutation tests from ClockstaRX on these data revealed that the first three PCs significantly describe variation in residual rates. Absolute 95% quantile outliers were taken for each of these PCs and used as three different outlier gene sets. These three sets were then compared to the outlier gene set identified by our new method using Mahalanalobis distances on the same pairwise *ω* distances. Fisher’s exact test was used to test if there is a significantly larger overlap between the ClockstaRX and the Mahalanobis gene sets than expected by chance (Figure S4).

#### Pathway enrichment analysis of outlier genes

We conducted a Kyoto Encyclopedia of Genes and Genomes (KEGG) enrichment analysis for the set of outlier genes identified, using the clusterProfiler R package.^67^ We report results involving outlier genes of *ω*, but analyses were also explored for outliers of *d*_N_, *d*_S_, overall molecular distances, and phylogeny-aware ClockstaRX PCs individually (results not shown). To assess background universe bias, we performed enrichment analyses using three different reference sets, (i-ii) the annotated gene sets of green sea turtle (*Chelonia mydas*; code *cmy*) and Chinese soft-shelled turtle (*Pelodiscus sinensis*; code *pss*) in KEGG database separately, and (iii) the combined gene set across 4 turtles species (*C. mydas*, *Chrysemys picta bellii*, *P. sinensis* and *Terrapene mexicana triunguis*). For the two KEGG-annotated organisms (*cmy* and *pss*), we converted outlier BUSCO genes to their corresponding NCBI Gene IDs as the forward gene set. For the multiple species background, the amino acid sequences were extracted from Orthodb 10^68^ database, and mapped to KEGG Orthologs (KOs) using KofamKOALA.^62^ Assigned KOs of outlier genes served as the forward gene set. Significant pathways were determined by applying a threshold of false discovery rate (FDR)–adjusted *P*-value <0.05. The significantly enriched pathways were visualized using dot plots, and we highlighted the outlier genes within these pathways using the pathview R package.^51^

### Quantification and statistical analysis

Statistical details of the various analyses conducted here can be found throughout the methods, results, figures and figure caption. Thresholds used to identify outlier genes and enriched pathways can be found in the methods. Descriptions of downstream analysis can be found in results, figures and figure legends. This applies to:

- correlation between divergence time and number of shared outliers
- correlations of interspecies trait and outlier-gene similarity.
- comparisons between phylogeny-free and phylogeny-aware methods.
