# Supplementary material for: Turtle genomic novelty is driven by the evolution of ecological robustness
Source: iScience. 2026 Feb 11;29(3):114975. doi: 10.1016/j.isci.2026.114975 (PMC12989854; doi:10.1016/j.isci.2026.114975)
Supplement: Document S1. Figures S1–S11 and Table S1 [file mmc1.pdf]

iScience, Volume *29*

Supplemental information

Turtle genomic novelty is driven
by the evolution of ecological robustness

Jule Drewalowski, Yuejiao Huang, Wessel Mulder, and David A. Duchêne

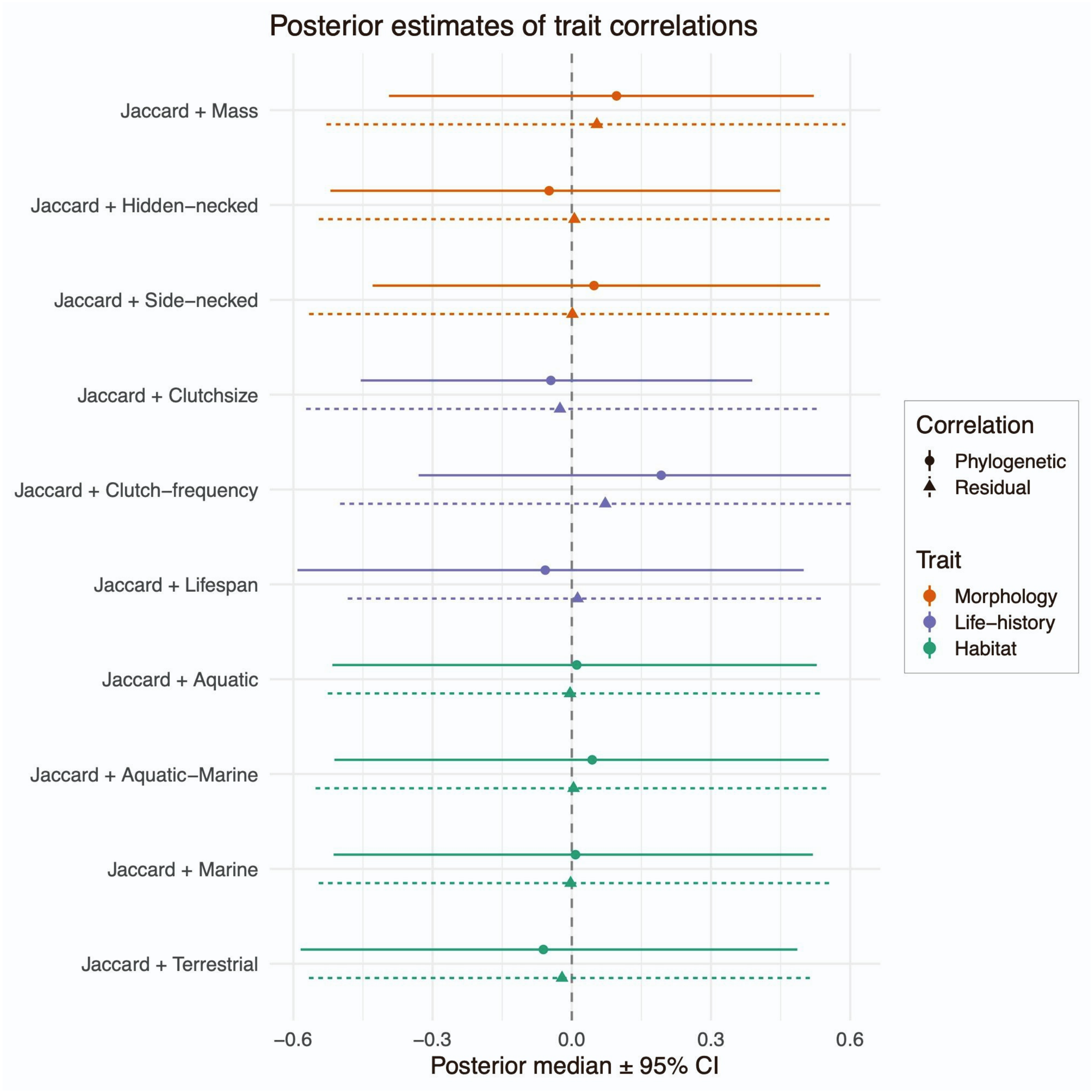

Supplemental Figure S1. Posterior estimates of phylogenetic and residual correlations between mean genetic uniqueness (Jaccard index) and ecological, morphological, and life-history traits in turtles. Points indicate posterior medians, and horizontal lines show 95% credible intervals. Circles represent phylogenetic correlations, while triangles represent residual correlations. Trait categories are shown by colour: morphology (orange), life history (purple), and habitat (green). All estimates have 95% credible intervals overlapping zero, indicating no significant association between genetic uniqueness and any of the measured traits after accounting for shared evolutionary history.

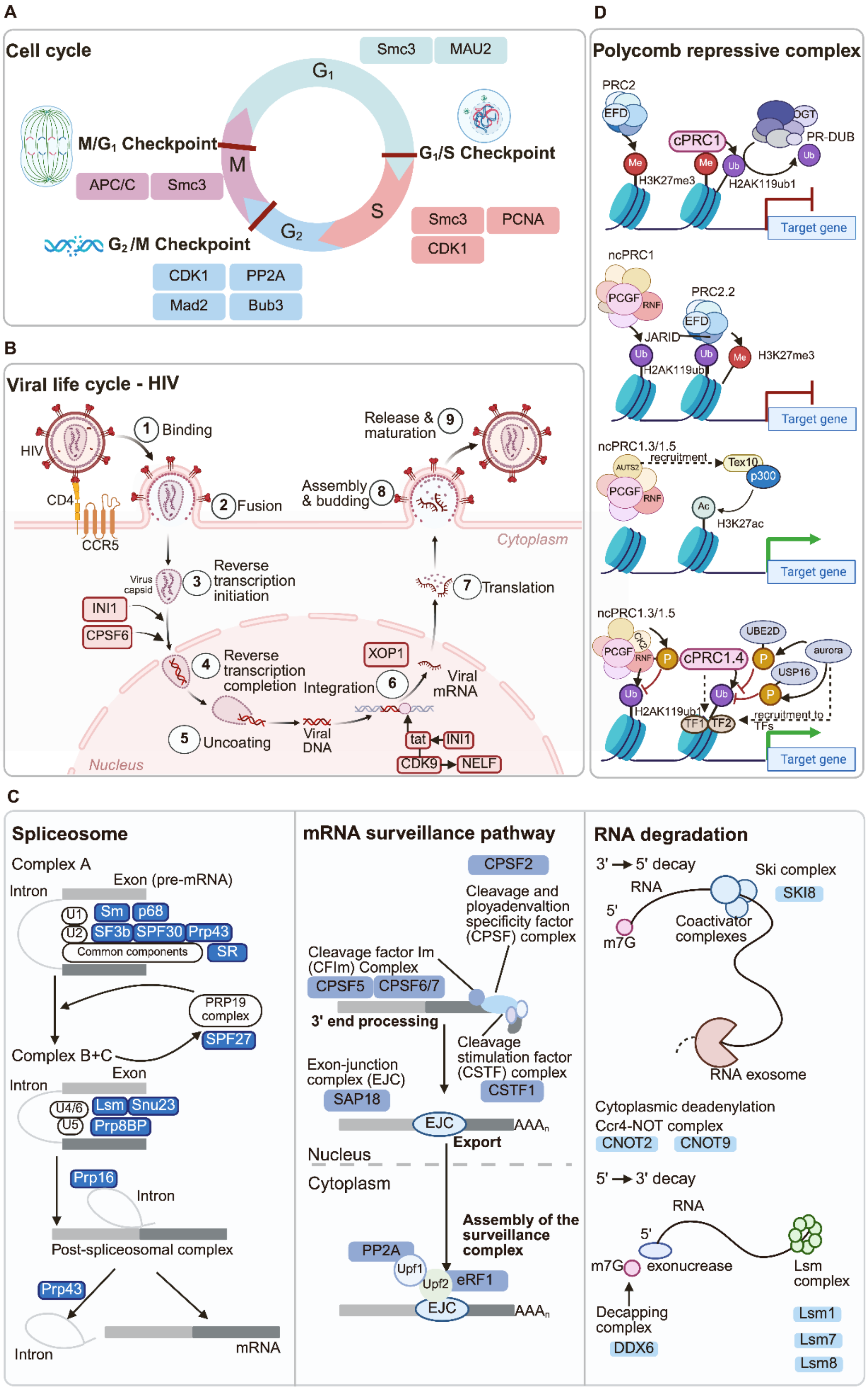

Supplemental Figure S2. KEGG pathways enriched in the outlier genes across all turtle taxa considered in this study. Pathways include the (A) cell cycle, (B) viral life cycle - HIV, (C) ribonuclear machineries, including spliceosome, mRNA surveillance and RNA degradation, and (D) polycomb repressive complex. Two large and complex enriched pathways are not shown,

including basal transcription factors and proteasome. The figure was created using Biorender templates, including "Cell Cycle Checkpoints Callout (Layout)" for panel (A) and "HIV Replication Cycle" for panel (B).

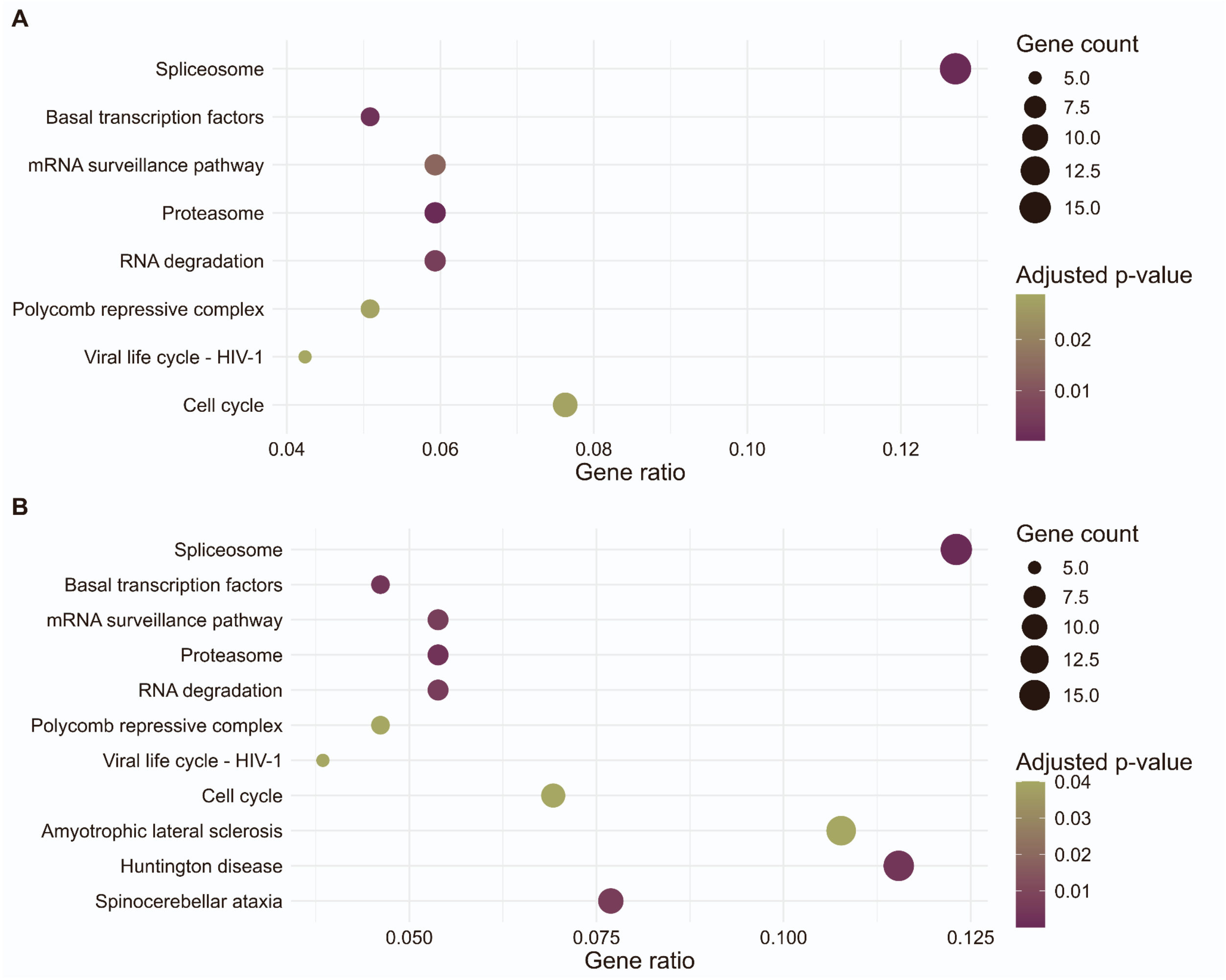

Supplemental Figure S3. KEGG pathway enrichment using alternative background gene sets. (A) Using KEGG-annotated Chinese soft-shelled turtle (pss) gene set as the universe background. (B) Using pooled gene sets from 4 turtle species as the background.

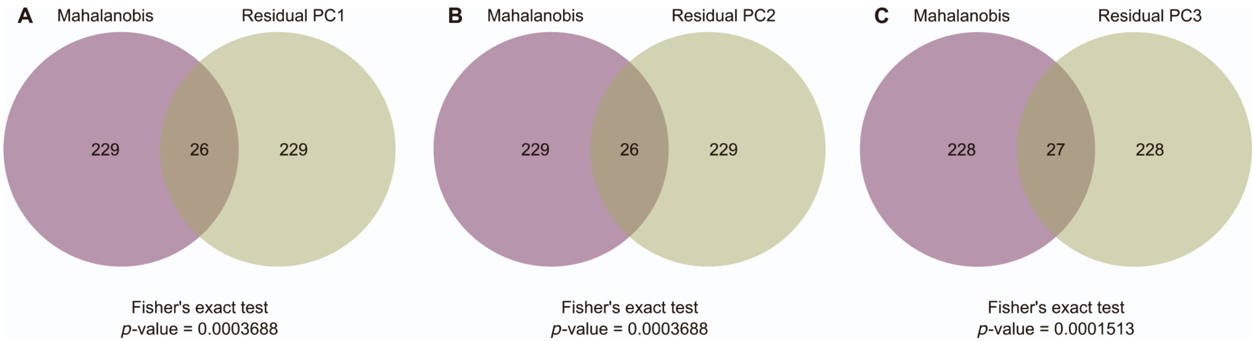

Supplemental Figure S4. Comparison of the phylogeny-aware method implemented in ClockstaRX against the phylogeny-free method based on pairwise distance matrices. Venn diagrams show the overlap of outliers identified using Mahalanobis distances under the method proposed here, compared to outliers identified using ClockstaRX PC1 (A), PC2 (B) and PC3 (C). Fisher's exact test *p*-values show that overlap in all three cases are significantly greater than expected at random, validating the new method. However, the absolute number of overlapping genes is small, and Mahalanobis distances identify different outliers than the phylogeny-aware method.

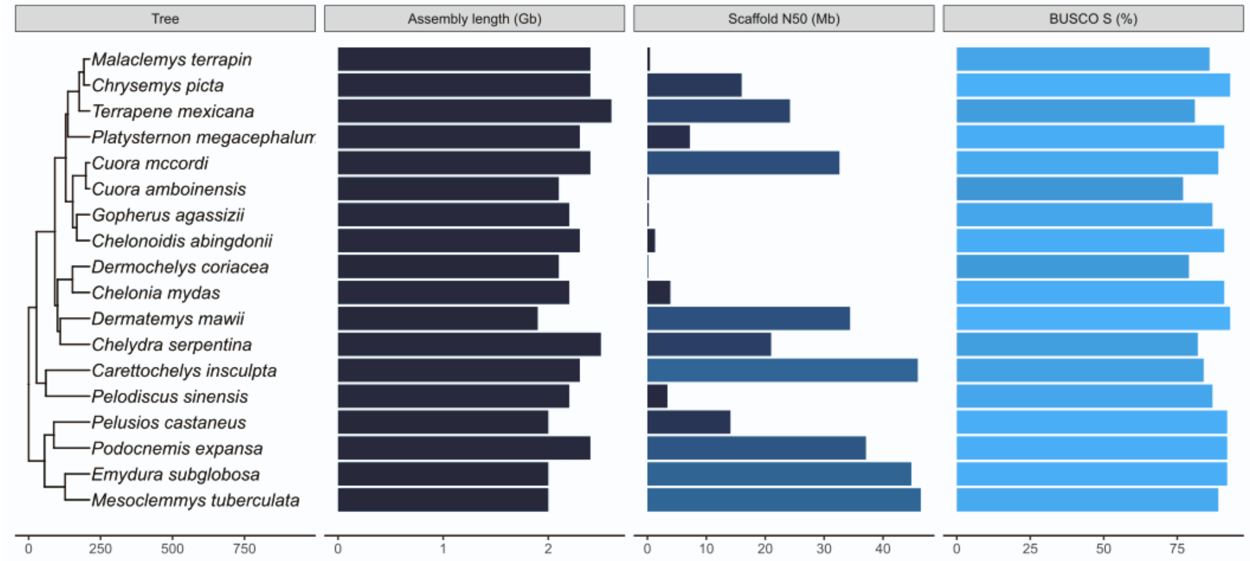

Supplemental Figure S5. Statistics about genomic assemblies across the turtle species considered in this study, based on Gable et al. (2022)[1]. While the Scaffold N50 shows varying levels of contiguity, BUSCO levels show generally high completeness in all genomes used.

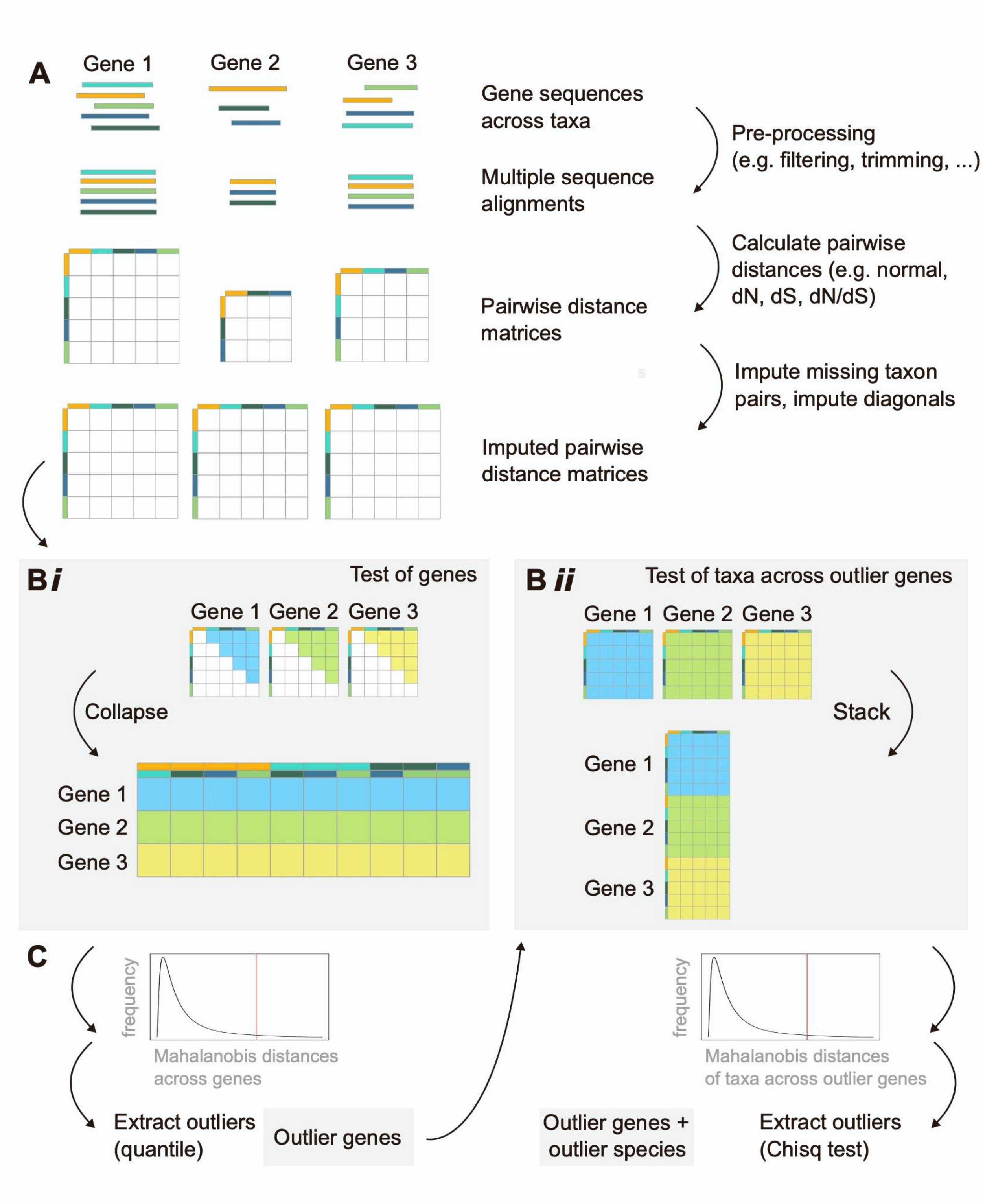

Supplemental Figure S6. Pairwise distance-based method of extracting outlier genes and taxa from phylogenomic datasets. (A) The workflow takes genetic pairwise distance matrices, here

calculated for either d_N, d_S, ω, and raw Hamming pairwise molecular distances across genes. Missing data are imputed to ensure matrices of equal size for each gene. These matrices are (B *i.)* collapsed to test genes for having outlier high divergence compared with the dominant genetic signal, and (B *ii.*) stacked across outlier genes to test combinations of genes and taxa for outlier high genetic distance. The approach uses the (C) Mahalanobis distances from the multivariate space where variables are either taxon pairs of individual taxa, accounting for any correlation structure across these variables.

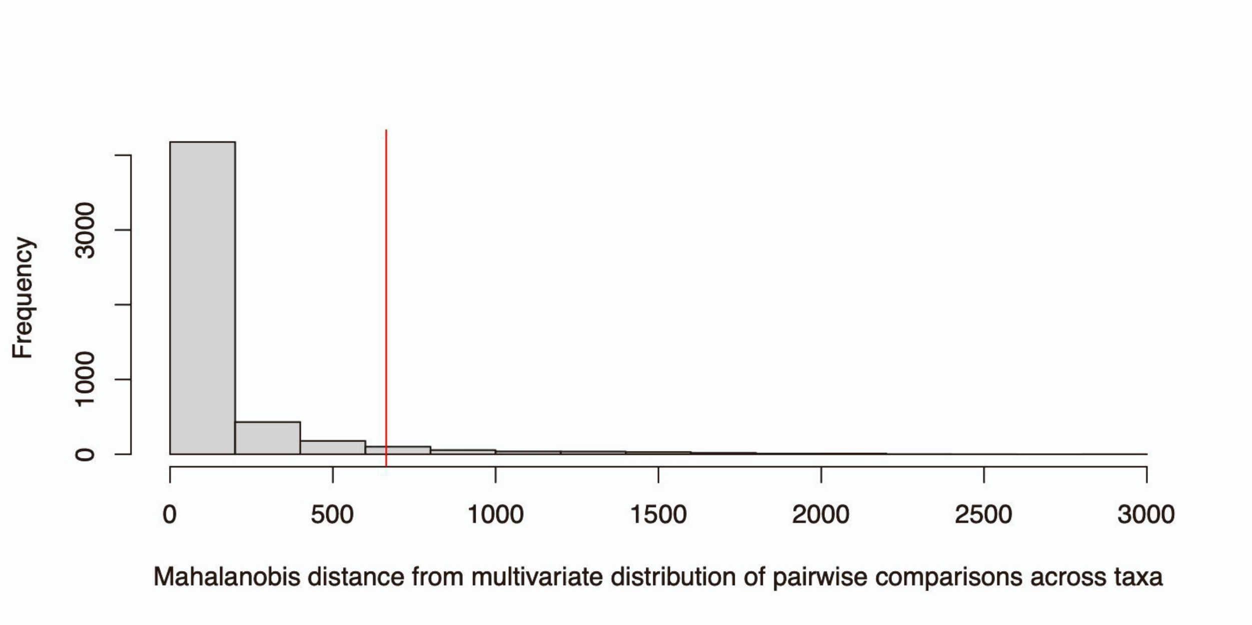

Supplemental Figure S7. Histogram of Mahalanobis distances in ω (d_N/d_S) across all genes. The red vertical line indicates the threshold value for the 95% quantile.

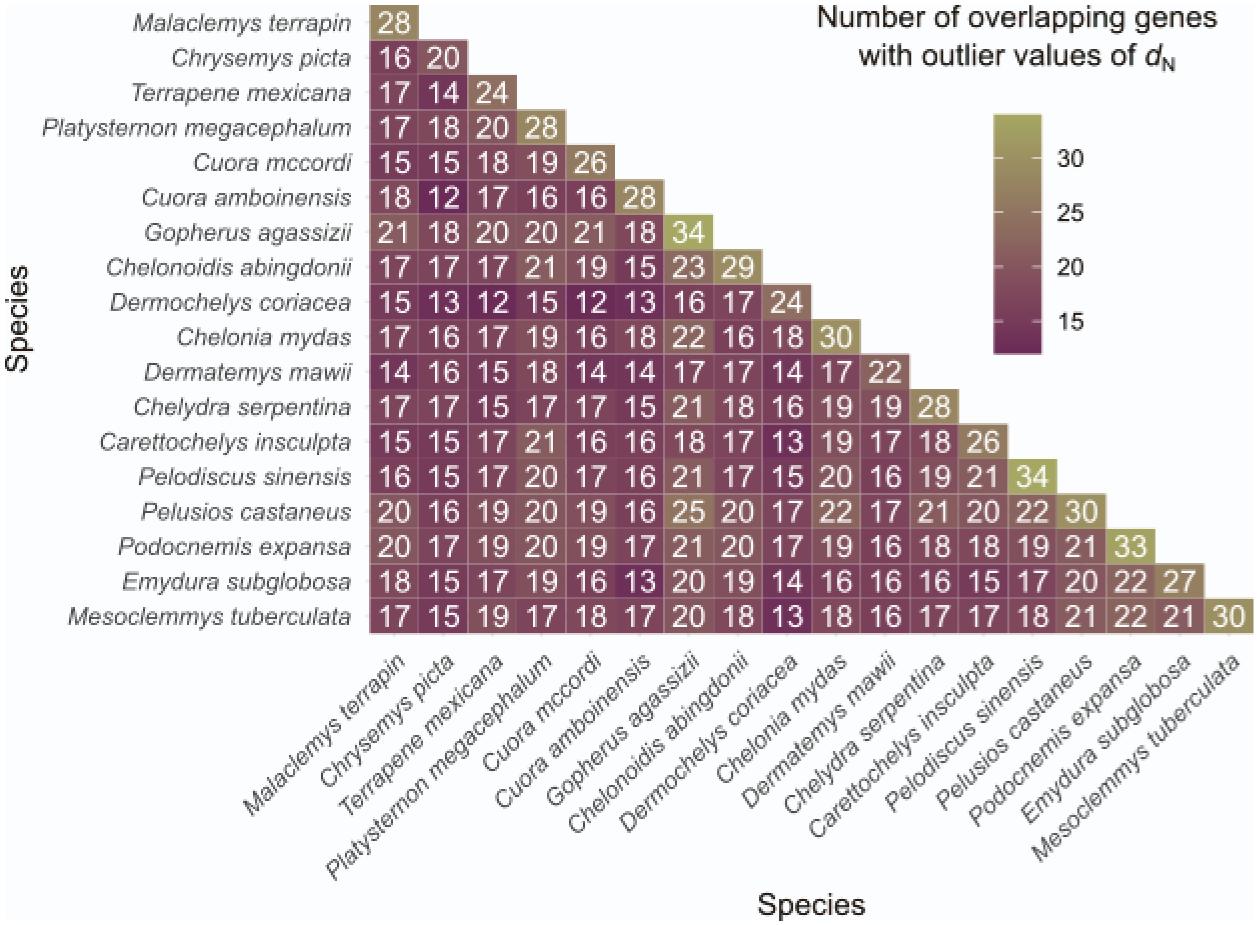

Supplemental Figure S8. Number of genes that overlap in having outlier high d_N among pairs of turtle taxa considered.

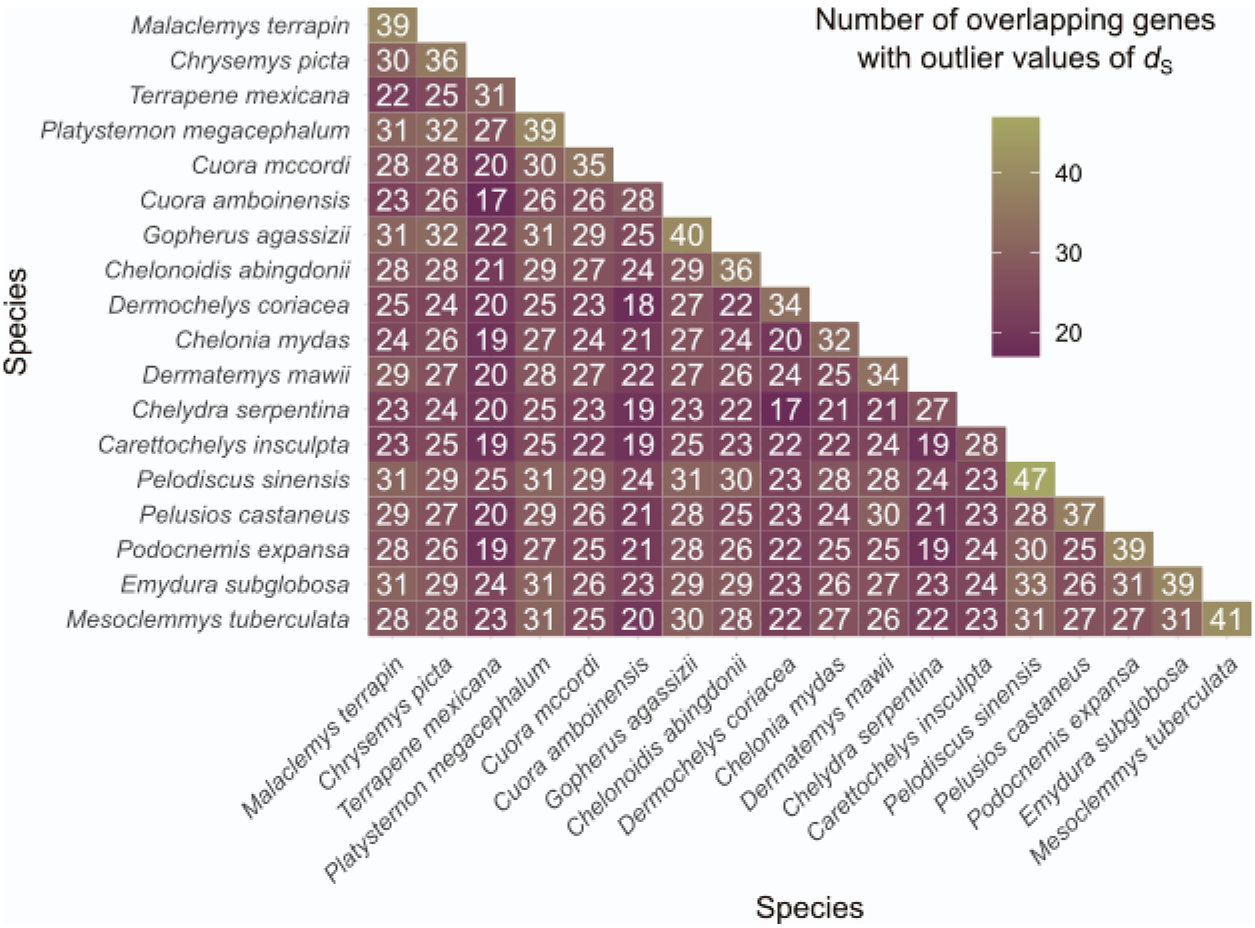

Supplemental Figure S9. Number of genes that overlap in having outlier high d_S among pairs of turtle taxa considered.

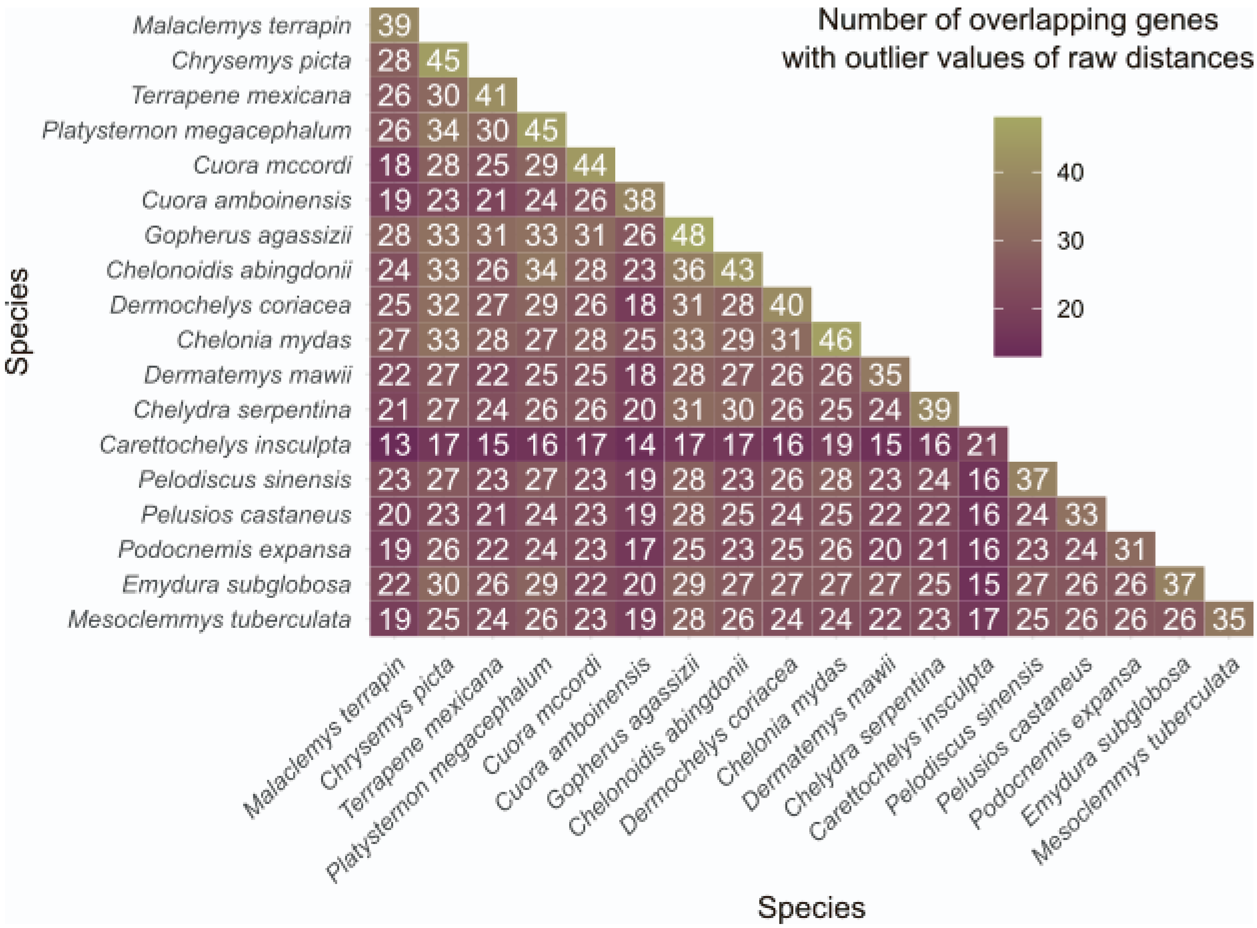

Supplemental Figure S10. Number of genes that overlap as having outlier high raw Hamming genetic distance among pairs of turtle taxa considered.

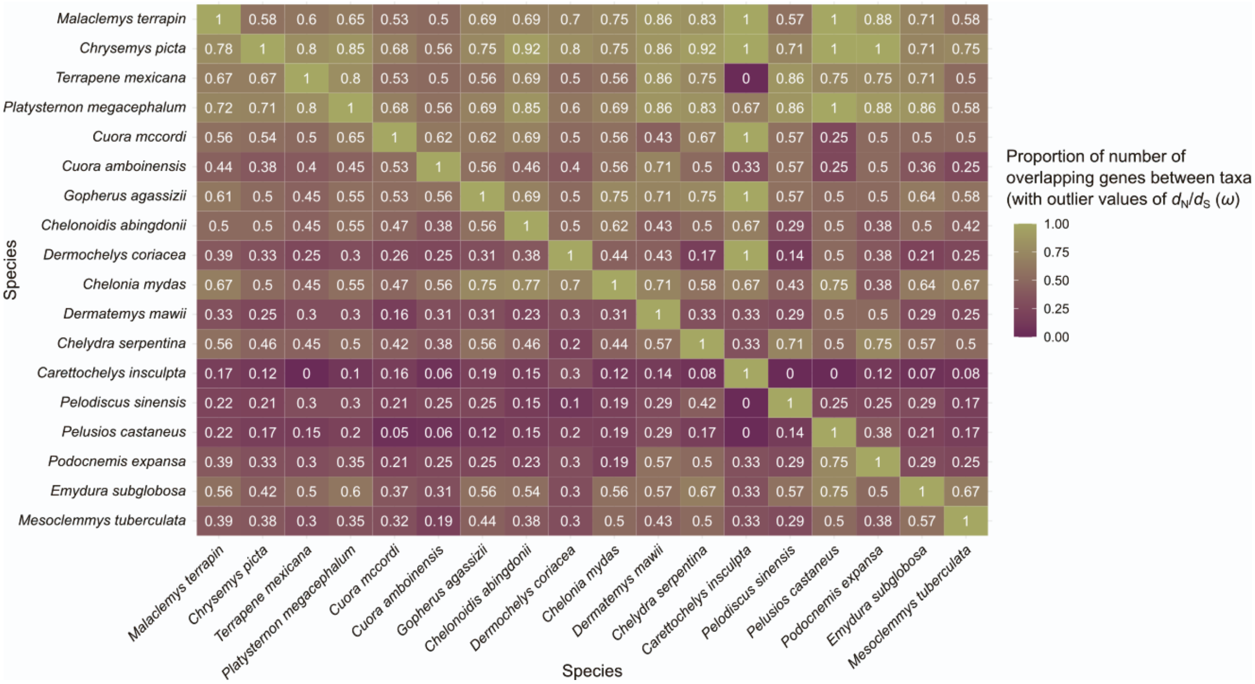

Supplemental Figure S11. Number of genes that overlap as having outlier d_N/d_S among pairs of turtle taxa considered, calculated as a proportion of the total number of genes for taxa across the vertical axis (upper triangle) and horizontal axis (lower triangle).

Supplemental Table 1. Full set of enriched functional terms and the contributing outlier genes across the species of turtles included in this study.

KEGG Category		Pathway	Pathway *p*-value	BUSCO ID	Gene symbol	Mahalanobis χ^2 *p*-value
Genetic Information Processing	Transcription	Spliceosome	2.83E-06	101949at32523	DDX5	3.34E-73
				165745at32523	RBM22	2.64E-177
				16602at32523	SF3B1	2.52E-192
				182549at32523	SNRNP40	2.14E-163
				251875at32523	SMNDC1	1.23E-92
				257507at32523	BCAS2	3.13E-171
				270377at32523	ZMAT2	4.58E-186
				279702at32523	SRSF5	1.46E-194
				290216at32523	SF3B6	6.16E-181
				293250at32523	SRSF3	1.42E-218
				294091at32523	PHF5A	3.44E-159
				294828at32523	LSM7	2.51E-294
				298204at32523	SNRPD1	0
				298305at32523	LSM8	7.88E-293
				30310at32523	DHX38	5.51E-111
				64249at32523	DHX15	1.10E-118
		Basal transcription factors	1.52E-03	194637at32523	GTF2B	2.01E-86
				219664at32523	GTF2F2	4.87E-218
				247336at32523	TAF8	2.16E-72
				259026at32523	TBPL1	3.00E-95
				292234at32523	GTF2A2	0
				305138at32523	GTF2H5	2.84E-186
	Translation	mRNA surveillance pathway	8.96E-03	116132at32523	CSTF1	8.16E-293
				141057at32523	ETF1	2.30E-185
				180491at32523	PPP2R5E	1.60E-110
				232639at32523	CPSF6	9.36E-173
				267603at32523	NUDT21	2.31E-195
				277586at32523	SAP18	7.60E-249
				58163at32523	CPSF2	6.81E-90

	Folding, sorting and degradation	Proteasome	4.27E-04	140221at32523	PSMC3	7.77E-123
				150509at32523	PSMC2	3.63E-116
				157171at32523	PSMD11	1.03E-163
				174675at32523	PSMD6	2.36E-204
				200165at32523	PSMD14	3.34E-143
				219728at32523	PSMD7	2.47E-199
				238978at32523	PSMA2	0
		RNA degradation	4.27E-03	107213at32523	CNOT2	8.63E-72
				128422at32523	DDX6	2.43E-253
				184192at32523	SKIC8	1.72E-107
				194996at32523	CNOT9	7.04E-206
				294828at32523	LSM7	2.51E-294
				298305at32523	LSM8	7.88E-293
				299214at32523	LSM1	2.99E-111
	Chromosome	Polycomb repressive complex	2.65E-02	142484at32523	EED	0
				178984at32523	DCAF7	2.18E-192
				224356at32523	PCGF3	8.78E-155
				23620at32523	USP7	2.12E-64
				25837at32523	OGT	7.45E-62
				283776at32523	MAX	2.05E-171
	Information processing in viruses	Viral life cycle - HIV-1	2.65E-02	166537at32523	SMARCB1	8.71E-221
				169435at32523	CDK9	9.40E-155
				232639at32523	CPSF6	9.36E-173
				24343at32523	XPO1	1.30E-124
				94920at32523	NELFCD	1.31E-78
Cellular Processes	Cell growth and death	Cell cycle	2.54E-02	109646at32523	ANAPC7	4.58E-94
				171646at32523	BUB3	1.01E-68
				180491at32523	PPP2R5E	1.60E-110
				192580at32523	CDK1	1.47E-115
				249342at32523	MAD2L2	4.67E-79
				256114at32523	PCNA	1.67E-82
				27867at32523	SMC3	5.11E-81
				83849at32523	MAU2	3.09E-206
				90752at32523	CDC23	1.17E-112

References

1. Gable, S.M., Byars, M.I., Literman, R., and Tollis, M. (2022). A genomic perspective on the evolutionary diversification of turtles. Syst. Biol. *71*, 1331–1347.
